# Three-dimensional traction technology and its application in mechanomedicine^☆^

**DOI:** 10.1016/j.mbm.2025.100131

**Published:** 2025-04-24

**Authors:** Xinman Chen, Chenyang Ji, Xi Liu, Ning Wang, Fuxiang Wei, Junwei Chen

**Affiliations:** a Key Laboratory of Molecular Biophysics of the Ministry of Education, Hubei Bioinformatics and Molecular Imaging Key Laboratory, Laboratory for Cellular Biomechanics and Regenerative Medicine, Department of Biomedical Engineering, College of Life Science and Technology, Huazhong University of Science and Technology, Wuhan, Hubei 430074 China; b The Institute for Mechanobiology, Northeastern University, Boston, MA 02115, USA; c Department of Bioengineering, College of Engineering, Northeastern University, Boston, MA 02115, USA

**Keywords:** 3D traction force, Traction force microscopy, Tumor microenvironment, Mechanomedicine

## Abstract

Endogenous forces generated by living cells are essential for biological processes and physiological functions of cells and tissues. Over the last several decades, numerous methods for detecting traction forces have been developed. Here we review these methods and discuss their respective strengths and limitations. Being able to reliably quantify tractions in living cells and tissues are critical in understanding how forces drive and regulate cell and tissue functions in physiology and diseases.

## Graphical abstract

**Figure:**
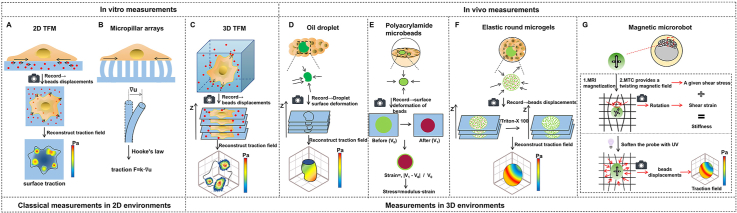

## 1 Introduction

**Fig. 1:**
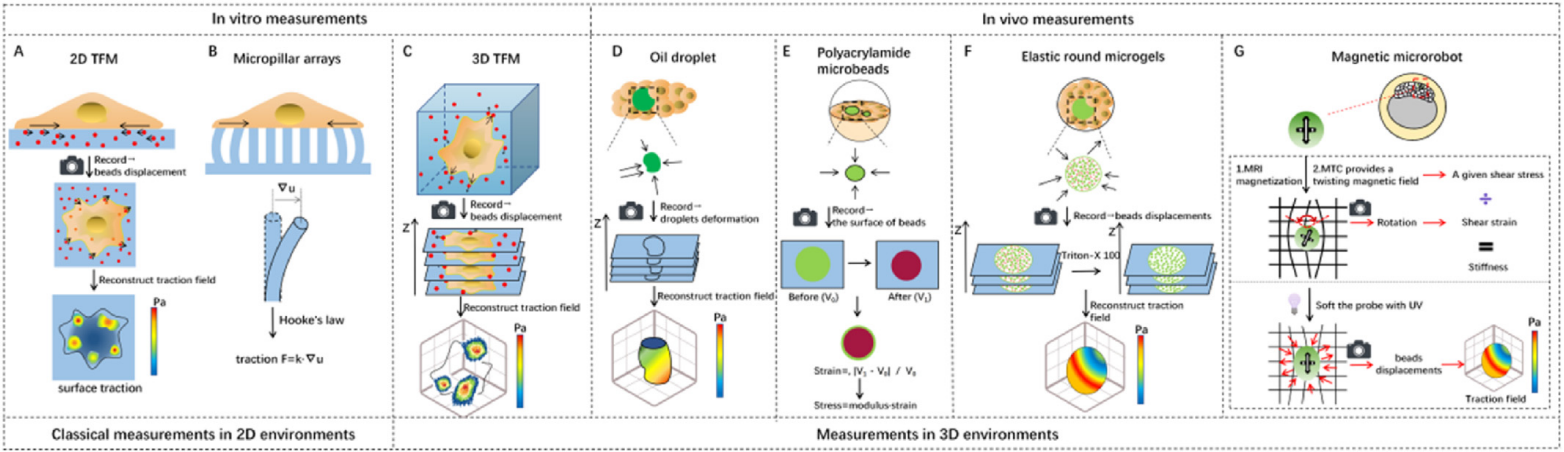
**Comparisons between different cell traction approaches**. A. Schematic of 2D traction force microscopy: Measuring cellular tractions using displacements of fluorescent marks on the surface of a two-dimensional material. B. Schematic of micropillar arrays: Measuring cellular tractions using elastic deformation of micropillar arrays. C. 3D traction force microscopy: Measuring cellular tractions using displacements of fluorescent marks in 3D. D. Oil droplet force sensor: Measuring cellular tractions using deformation of oil droplet surfaces in tissues. E. Polyacrylamide microbeads: Measuring cellular tractions using deformation of polyacrylamide microbeads in cellular tissue. F. Elastic round microgel force probe: Measuring cellular tractions using 3D displacements of fluorescent marks in elastic microspheres. G. Magnetic microrobot: Magnetic materials encapsulated in an elastic gel for stiffness and traction measurements.

Increasing evidence indicates that mechanical forces are crucial in regulating processes in development, physiology, and disease.^1^, ^2^, ^3^, ^4^, ^5^ For instance, anisotropic forces in Drosophila embryos drive tissue elongation;^6^ forces play significant roles in stem cell differentiation^7^^,^^8^ and in organized germ layer patterning in mammalian embryogenesis.^9^ Mechanical forces are also important in cancer progression.^10^, ^11^, ^12^, ^13^ Consequently, quantifying the mechanical forces generated by living cells is essential for understanding the mechanisms of physiology and diseases. Several key methods have been developed to estimate mechanical forces in living cells on 2D surfaces, in 3D matrices, and even in living tissues. In this review, we highlight these technologies (Fig. 1) and their applications in quantifying forces and moduli in cells and *in vivo*. We also discuss the application of mechanobiology to medicine, i.e., mechanomedicine. Mechanomedicine uses mechanobiology-based principles and technologies at molecular, cellular, tissue, and whole-body scales *in vivo* to the diagnosis, treatment, control, and cure of various human diseases.

## 2 2D traction force quantification

### 2.1 2D traction force microscopy (TFM)

In the early 1980s it was observed that fibroblasts on silicone rubber substrates generate wrinkles on flexible rubber substrates.^14^ This observation suggests that these non-muscle cells generate tractions. However, silicone rubber had limited practical use due to its opacity. This opacity made it difficult to quantify tractions from adherent cells.^15^^,^^16^ By the late 1990s, a polyacrylamide (PA) hydrogel method was developed as superior substrates for cellular traction force measurement on 2D surfaces, thanks to their optimal mechanical and chemical properties and optical transparency, facilitating standard fluorescence microscopy assessment of cell-induced deformations.^17^ In 1999, a method of seeding the cells on PA hydrogels to quantify traction forces of adherent cells was developed using inverse operations via the Boussinesq theory, thereby establishing TFM technology.^18^ This TFM method on the PA substrate used Green's functions of the elasticity equations, similar to a previous paper in 1996 by the same first author involving thin liquid-supported silicone films^19^ (Fig. 1A). However, this method of quantifying tractions is limited by detection errors of fine protrusions and extensions at the cell periphery. Using the same PA hydrogel substrate, an alternative method of quantifying the tractions from the displacement maps was developed using Fourier transform traction microscopy that was more robust and dependable in tractional stress quantification. This technology enables the calculation of cellular traction stress by tracking the displacements of fluorescent nanobeads within the PA hydrogel and by finding the solution of the relationship between displacements and tractions into Fourier space, yielding sensitive and quantitative data that elucidate the dynamics of force generation within cells^20^, ^21^, ^22^ (Fig. 1A). Using microfabricated arrays of elastomeric micropillars, a different approach of quantifying tractions and isolating forces was developed later^23^ (Fig. 1B). In comparison to the micropillar method, the TFM method has the advantage of making it easy to apply the PA gel without using the sophisticated microfabrication process and being more physiologically relevant regarding the pore sizes of the matrix (∼100 ​nm). However, the forces are not isolated in the TFM method.

2D traction force measurements are still frequently employed nowadays, and their high resolution allows for the quantification of forces between cells and cell–matrix interactions. This information has shed light on cell development, mechanical force transmission, normal physiological processes, and disease processes.

However, cells also generate stresses in the z-direction, perpendicular to the x-y plane, even in a two-dimensional environment. To address this issue, 2.5-dimensional TFM was developed, enabling the detection of both normal and tangential forces.^24^^,^^25^ A 2.5D TFM with high spatiotemporal resolution was developed to simulate 3D cellular traction forces on a 2D plane.^26^ This technique revealed that the center of focal adhesions produces shear traction, with the distal (near the cell border) and proximal (near the cell body) ends generating upward and downward traction, respectively. Building on this approach, a linear polarization modulation (LPM) method was designed,^27^ utilizing individual nitrogen vacancy centers in nanodiamonds as fluorescent markers. This strategy can measure both translational and rotational motions induced by shear forces.

### 2.2 DNA force probe

Investigating mechanotransduction at the molecular level requires sensitive force-sensing techniques beyond the capabilities of traction force microscopy (TFM), which is limited in resolving forces at the nanonewton scale.^28^ To measure the subtle forces exerted by individual cellular receptors or proteins, especially during ligand-receptor interactions, advanced force-sensing probes based on molecular springs have been developed. These probes can reversibly extend in response to piconewton forces, revealing details of mechanically sensitive cellular processes.^29^ Two primary methodologies for tension probes are tension gauge tether (TGT) and molecular tension fluorescence microscopy (MTFM).

DNA-based molecular probes have revolutionized the measurement of single-molecule biomolecular interactions.^30^ Early Tension Gauge Tethers (TGT) probes identified piconewton forces in ligand-receptor binding and revealed rupture forces of 40 ​pN for integrin-ligand bonds and less than 12 ​pN for Notch receptor activation but it was difficult to detect ligand location.^31^

To address this, fluorescent TGT probes were created in 2015 to visualize ligand distribution and cellular responses.^32^ However, these probes suffered from fluorescence quenching, leading to potential measurement inaccuracies. Integrated Tension Sensors (ITS) and Quenched Tension Gauge Tethers (qTGT) were then engineered to fluoresce only upon DNA tether rupture, enabling real-time, multi-level tension analysis.^33^^,^^34^ However, the probes were limited to single-use due to irreversible DNA breakage.

Molecular Tension Fluorescence Microscopy (MTFM) and Molecular Tension Probes (TPs),^35^ featuring DNA hairpins with fluorophore-quencher pairs, were developed. These probes fluoresce upon unfolding under specific forces, revealing non-uniform traction force distributions within focal adhesions.^36^^,^^37^ A Force-Chrono probe was developed that can measure the magnitude, duration, and loading rate of forces at the single-molecule level within living cells.^38^ Recently, a method of quantum-enhanced diamond molecular tension microscopy (QDMTM) was developed for measurements of cellular adhesion forces.^39^ The key to this method is to correlate nitrogen-vacancy spin relaxation time *T*_1_ with force-induced polymer stretching, using the worm-like chain model to extract cellular forces from *T*_1_ mapping. While these innovations have improved our understanding of cellular mechanotransduction and mechanosensitivity, these force probes are limited by the inability to quantify the directions of the tractional stresses.

## 3 3D traction force quantification

### 3.1 3D TFM

In comparison to 2.5D TFM that enables the detection of 3D tractions for cells on 2D planar surfaces,^40^ the 3D TFM is able to quantify both normal and shear stresses for cells cultured within the 3D tissues. By tracking fluorescent bead displacements and applying linear elasticity theory, this method allows quantitative measurement of cellular forces in 3D microenvironments^41^ (Fig. 1C). Furthermore, building upon earlier linear methodologies, a nonlinear finite element framework was introduced to address limitations imposed by linear material assumptions. This approach employed multiple test force magnitudes to establish a weighted superposition model of displacement responses, integrated with hyperelastic constitutive equations (e.g., Mooney-Rivlin model), substantially reducing force field reconstruction errors.^42^ Experimental validation revealed that utilizing three test forces (1 ​nN, 10 ​nN, 100 ​nN) decreased average errors from 115 ​% in linear models to 17 ​%, with notable improvements in low-force regimes. Subsequent refinements targeted highly nonlinear biopolymer networks (e.g., collagen gels) through a semi-affine continuum model, combining local non-affine fiber deformations (buckling, stretching) and macroscopic affine strains to formulate a constitutive equation capturing matrix nonlinearity.^43^^,^^44^ This framework enabled dynamic quantification of traction forces in breast cancer cells migrating through collagen gels of varying concentrations, uncovering strong correlations between contractility, morphological plasticity, and migratory persistence. Collectively, 3D cellular traction force microscopy has evolved from linear simplifications to nonlinear analyses, bridging idealized models with physiologically complex microenvironments to enhance both precision and biological relevance.^45^ A high-resolution 3D Traction Force Microscopy (TFM) algorithm, employing a large deformation formulation, enables the quantification of cellular displacement fields with an improved resolution.^46^ This approach has advanced understanding of cellular mechanosensing and mechanotransduction. Emerging techniques like traction force optical coherence microscopy (TF-OCM) now offer label-free, high-throughput volumetric imaging, facilitating traction analysis in dense cell collectives (e.g., tumor spheroids).^47^ Concurrently, inverse hyperelasticity frameworks have improved traction mapping in native extracellular matrices by accounting for geometric and material nonlinearities.^48^^,^^49^

3D Traction Force Microscopy (TFM) facilitates *in vitro* mechanical interaction studies across scales, ranging from cellular to tissue levels and spheroid models, and simulates diverse *in vivo* environments for investigating disease progression, wound healing, angiogenesis, and related phenomena. The selection of the optimal methodology is guided by the specific requirements of each investigation. Key challenges include elucidating the intrinsic relationships of nonlinear materials employed to mimic physiological conditions and recognizing that cell-generated forces may not uniformly result in matrix deformation. A recent report suggests that measurements of local tissue fiber orientation and density can be used as a qualitative proxy for changes in traction forces,^43^ though issues remain for *in vivo* conditions, where real-time force measurement is hindered by tissue opacity, challenges in quantifying fiber orientation and density, the inability to control boundary conditions, and the difficulty in knowing the stress-free conditions to precisely quantify the magnitudes of 3D stresses *in vivo*.

### 3.2 3D *in vivo* force sensors

The methods described above offer a straightforward three-dimensional model for the *in vitro* measurement of mechanical forces operating on cells or tissues. Although researchers attempt to closely replicate the *in vivo* cellular environment by varying materials, they remain too homogeneous and streamlined in comparison to the highly dynamic and variable matrices found in living tissues. The viscoelastic behaviour exhibited by the extracellular matrix reinforces the importance of the dynamic properties of biomaterials.^50^ Moreover, the three-dimensional model offers limited space for cell movement, and the impact on their physiological state is unknown.

For *in vivo* applications of traction technology to facilitate quantitative analysis of dynamically changing cellular tractions in the dynamic environments of the organism, new strategies must emerge. A fast-developing technology that promises to meet this requirement is to create mechanically stable materials as sensors embedded in the actual *in vivo* conditions,^51^ instead of analog substrates.

Notably, 3D *in vivo* force sensors offer several advantages over traditional 2D or 3D traction force microscopy, including non-invasiveness, real-time monitoring, and local measurement capabilities. They are particularly suited for complex 3D systems where traditional methods fail to capture the intricate spatial and temporal dynamics of force distribution.

3D force sensors also provide high-resolution measurements, allowing for the detection of tiny changes that could be missed in bulk measurements. Furthermore, their small size enables targeted measurements within confined spaces, such as the interior of tissues or around individual cells, providing insights into local biomechanical properties that are critical for understanding cellular behavior and tissue function.

A brief overview will be given of the various materials used for these sensors, their force measurement capabilities, and their roles and applications *in vivo*. These approaches aim to bridge the gap between *in vitro* and *in vivo* models, offering a more realistic representation of the complex physiological environment cells experience within living organisms.

#### 3.2.1 Oil microdroplet force probes

A cell-sized fluorescent oil microdroplet force probe was developed that binds to cell adhesion molecules.^52^ Once the microdroplets are introduced to the cells, the nearby cells attach to the droplet surface and apply stresses, leading to droplet deformation. Local stresses can be estimated from the normal deformations using Laplace's equation (Fig. 1D). This is the first report of a method that can quantify local traction variations in living embryonic tissues in situ. Under various experimental setups, different surfactants can be added to the oil droplets to adjust the degree of deformation to suit different mechanical microenvironments. Recently the development of double emulsion droplet sensors enables in situ osmotic pressure measurements within 3D multicellular systems, including living embryonic tissues.^53^ These sensors reveal balanced pressures within the zebrafish embryo and significant differences from external environments, shedding light on osmotic pressure regulation in development. The oil microdroplet technique primarily quantifies anisotropic mechanical stresses (both compressive and tensile) generated by cells within living embryonic tissues. This method enables in situ mapping of stress anisotropy at cellular scales, revealing how spatial variations in mechanical forces drive morphogenetic events such as tissue folding and cell rearrangement during organogenesis. Yet, this method has limitations. Because oil is an incompressible liquid and there are no volume changes of the oil droplet under stress, the oil droplet method is limited by not being able to detect the hydrostatic stress, in addition to the possibility that the physical and chemical properties of the surfactant on the oil droplet surface might be altered by the living tissues. These limitations can be overcome by using elastic polymers instead of oil and non-surfactant surface material in the force probe.

#### 3.2.2 3D elastic force sensors

The limitation of the incompressible oil droplet force probe can be overcome by using elastic materials as deformation reporters. The elastic, mechanically explicit functionalized polyacrylamide (PAA) microbeads serve as another force sensor to assess isotropic stress. By measuring the probe's volume changes and incorporating those changes into various areas inside the cell mass (e.g., stresses in different regions along the radius of the tumor sphere), the mechanical stress distribution in three-dimensional tissues can be assessed without a quantitative stress distribution on the surface of the microbeads (Fig. 1E). It is ideal for examining the active response of cells to various external force situations because of its elastic qualities, which allow external mechanical forces to be applied (for example, raising osmotic pressure using dextrose).^54^^,^^55^ However, since the real force produced by a cell is three orders of magnitude smaller than the load applied externally, it is necessary to construct soft polyacrylamide microspheres to quantify cellular forces. The material's modulus of elasticity can be altered by altering the ratio of acrylamide to bis-acrylamide, making it possible to produce sensors with a broad range of moduli. However, polyacrylamide has the propensity to depolymerize into acrylamide molecules and produce biotoxicity when PA gels are very soft (e.g., at 0.1–1 ​kPa). Micro-spheroid models utilize hydrogel-based force sensors to measure both isotropic and anisotropic stresses within cell-dense tissues. These models have demonstrated an ability to detect subtle force variations exerted by cells on their microenvironment, providing novel insights into cellular sensing and response to mechanical stimuli.^56^ A study incorporates chain-terminated fluorescent monomers into the polymer backbone to partially break the polymer network and achieve reduced stiffness, which enables the measurement of stresses as low as 10 ​Pa.^57^ Quantification of cellular isotropic and anisotropic strains is made possible by quantification of compressibility and elastic modulus of the microbeads.^58^

**Fig. 2:**
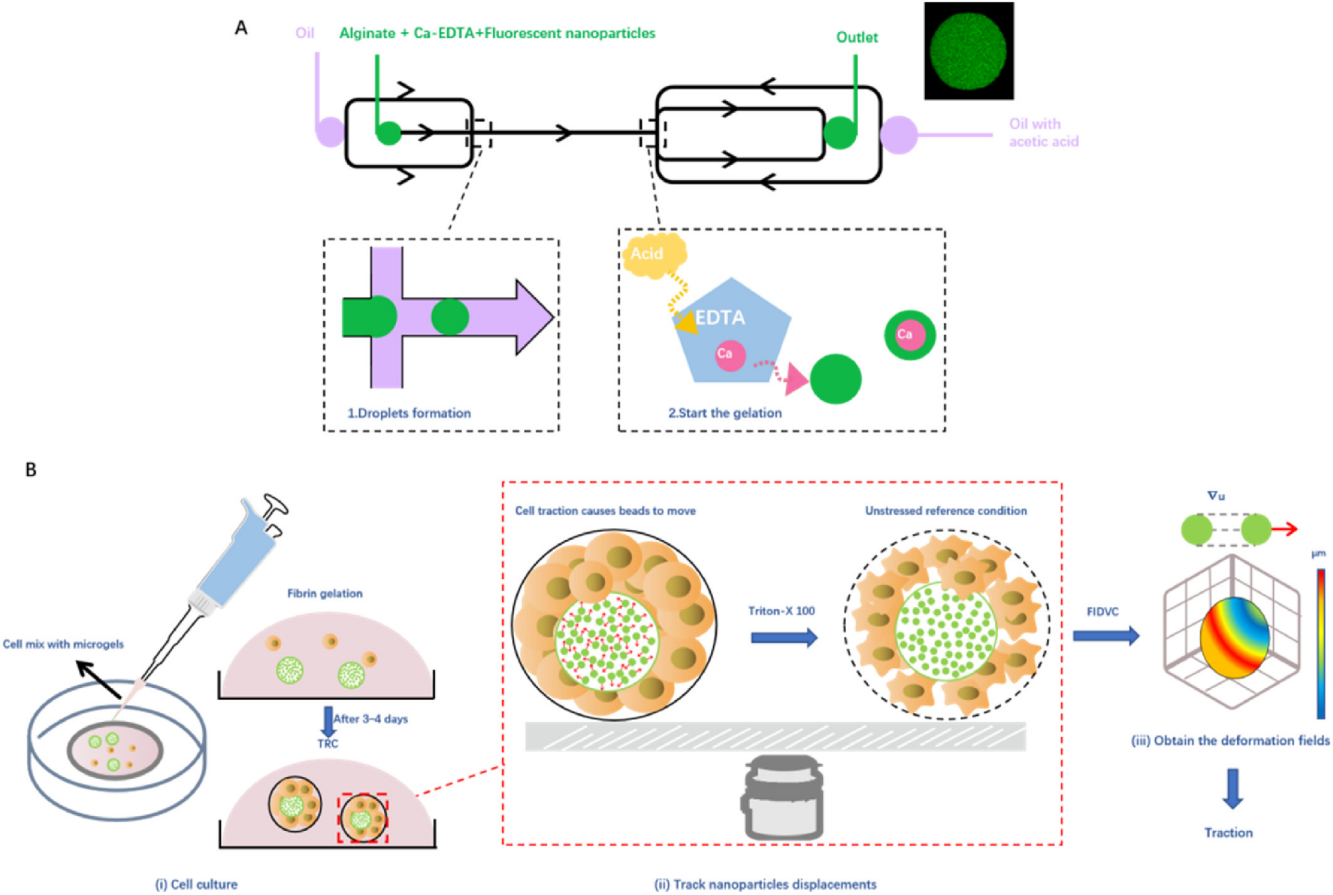
**3D elastic force probes quantify traction forces in a cancer cell colony**. A. Fabrication of microgels by a microfluidic device. B. (i) Tumor cells are cultured within 3D gels together with the elastic force probes labeled with fluorescent nanoparticles. (ii) Cell-generated forces deform the force probe, and the resulting displacement fields of fluorescent nanoparticles are measured. (iii) Traction forces are calculated from the displacement fields and the known modulus of the force probe. This figure is modified from Reference 59.

A more sophisticated in-vivo approach is essential for quantifying the forces at play during tissue development. The Elastic Round Microgel (ERMG) method (Figs. 1F and 2) addresses the need for comprehensive measurement of compressive, tensile, and shear stresses, allowing for dynamic monitoring of traction forces *in vivo*.^59^ This method utilizes microfluidic devices to create round alginate microgels with diameters of 25–30 ​μm, which are monodisperse, homogeneous, and elastic. Alginate is non-toxic and has elastic shear moduli ranging from 0.5 to 1.2 ​kPa, which can be adjusted by varying alginate concentration. By coupling sodium alginate with RGD (Arg-Gly-Asp) for cell adhesion and cross-linking with calcium ions, gelation is achieved through pH modulation, enhancing the microspheres' mono dispersion for *in vivo* measurements. The microgels are filled with 200 ​nm fluorescent nanoparticles, serving as markers for cell-induced deformation. The displacement field of these nanoparticles is analyzed using the fast iterative digital volume correlation (FIDVC) method. A robust three-dimensional strain and stress computation strategy is developed, utilizing a stress-free state via killing the cells with Triton X-100. Notably, this method reveals that compressive traction forces are generated at focal complexes, underscoring the importance of multi-dimensional traction measurement and the limitations of *in vitro* methods. By injecting microgels into early zebrafish embryos, this technique enables the quantification of 3D traction forces in living tissues, facilitating the examination of subtle spatial and temporal force changes during development. Quantified tractional stresses in multiple locations simultaneously reveal traction heterogeneity during early embryonic development in vertebrate. 59 Another approach for measuring cellular forces is by the introduction of hyper-compliant microparticles (HCMPs) for monitoring microscale cellular forces during mesenchymal condensation.^60^ These findings highlight the significance of sensor compliance in detecting minute changes in the mechanical microenvironment, with HCMPs offering a sensitive platform for spatiotemporal force measurements. In a parallel development, intravital mechano-sensory hydrogels (iMeSHs)^61^ have been bioprinted directly into the developing neural tube of chick embryos, facilitating the measurement of forces with micrometer-level resolution. This approach underscores the potential of *in vivo* force sensing, which could yield unprecedented understanding of the mechanics involved in developmental processes.

In another study, the selection of force sensors for in situ measurement of neotissue microenvironments was explored. The physical characteristics of force sensors, including size, elastic modulus, and surface coating, were varied to assess their impact on measurement fidelity. It was found that sensors with a diameter >20 ​μm and a modulus of 0.2 ​kPa provided the highest measurement fidelity. Protein coatings, such as collagen and N-cadherin, influenced the directionality of applied forces, allowing for tensile forces to be exerted on the sensors.^62^

#### 3.2.3 FRET-based force sensor

FRET (Fluorescence Resonance Energy Transfer)-based force sensors have become a valuable tool for measuring tensions in various biological systems for deformations that are in the order of several nanometers and forces that are in the range of tens of picoNewtons (pNs). For example, vinculin FRET force sensors^63^ and talin FRET force sensors^64^ have been developed to quantify the force transmission at the focal adhesions and reveal regulation dynamics and mechanosensitivity of focal adhesions. The actinin tension sensor ActTS-GR, a non-invasive FRET-based tool,^65^ measures tension in living cells and embryos by detecting changes in the distance between mCherry and EGFP fluorophores connected by a spider silk protein. A FRET-based sensor has been developed to measure E-Cadherin tensions in Drosophila tissues, facilitating both *in-vivo* and ex-vivo studies.^66^ This advancement enables the real-time visualization of dynamic cytoplasmic forces. The application of FRET-based force sensors has been expanded to investigate force transmission across the mammalian nuclear envelope *in vivo*.^67^ Additionally, these sensors have been employed to elucidate force redistribution in clathrin-mediated endocytosis.^68^ Collectively, FRET-based force sensors have emerged as a valuable resource for examining mechanical dynamics within live cells, offering insights into force transmission and tension across diverse biological systems. However, the maximum distance for efficient energy transfer is only ∼10 ​nm for FRET. In addition, one needs to use a pair of fluorophores with large spectral crosstalks, limiting its dynamic ranges in imaging. By developing reversible interaction of fluorescent proteins with a fluorescently labeled HaloTag with small spectral crosstalks,^69^ researchers are able to design FRET biosensors with wide dynamic ranges and convert these FRET biosensors into intensiometric and fluorescence lifetime-based sensors in the far-red range to improve their potential for deep tissue imaging.^70^

#### 3.2.4 Force sensor for subcellular force transport structures

In contrast to cell clusters or tissues, another method primarily focuses on detecting subcellular force transport structures. This particle-based force sensing technique is specifically designed for high-resolution studies of ligand-dependent cellular interactions. Monodisperse spherical hydrogel particles are produced using a homogeneous pore-size porous glass (SPG) membrane emulsification process,^71^ closely resembling the size and stiffness of target cells (0.3–10 ​kPa). A reference-free calculation method is presented that does not rely on tracer particles; instead, it iteratively minimizes a cost function comprising residual tractive force in the traction-free region and elastic energy, employing a fast spherical harmonic-based approach to infer tractive forces from measured particle shapes. This technique enables the analysis of the kinetics of immunological synapses with T cells and variations in normal and shear traction during phagocytosis.

#### 3.2.5 3D sensors for force and stiffness in 3D tissues

Cell tractions and cell mechanical properties appear to be two important parameters in cell mechanics. Principles. For single cells on 2D surfaces, stiffness and traction are shown to be proportional. However, in multicellular tissues in 3D, the relationship between the two is not clear. The simultaneous measurement of these two quantities is essential for a comprehensive understanding of the impact of mechanical properties on biological activities. However, to quantify the forces, the sensor must be soft and to quantify stiffness the probe must be stiff. Therefore, it is difficult to quantify these two parameters using the same probe at the same site of the tissues.

A prominent technique utilizes biocompatible, magnetically responsive ferrofluid microdroplets as local mechanical actuators,^72^ facilitating the direct *in vivo* quantification of spatially varying mechanical properties within developing three-dimensional tissues and organs. This approach has demonstrated that during vertebrate body elongation, such as in the development of zebrafish tailbuds, tissues exhibit viscoelastic behavior, with decreasing stiffness and increasing fluidity towards the posterior region. However, this method only reports data on small oscillations in normal tractions and lacks other local information.

**Fig. 3:**
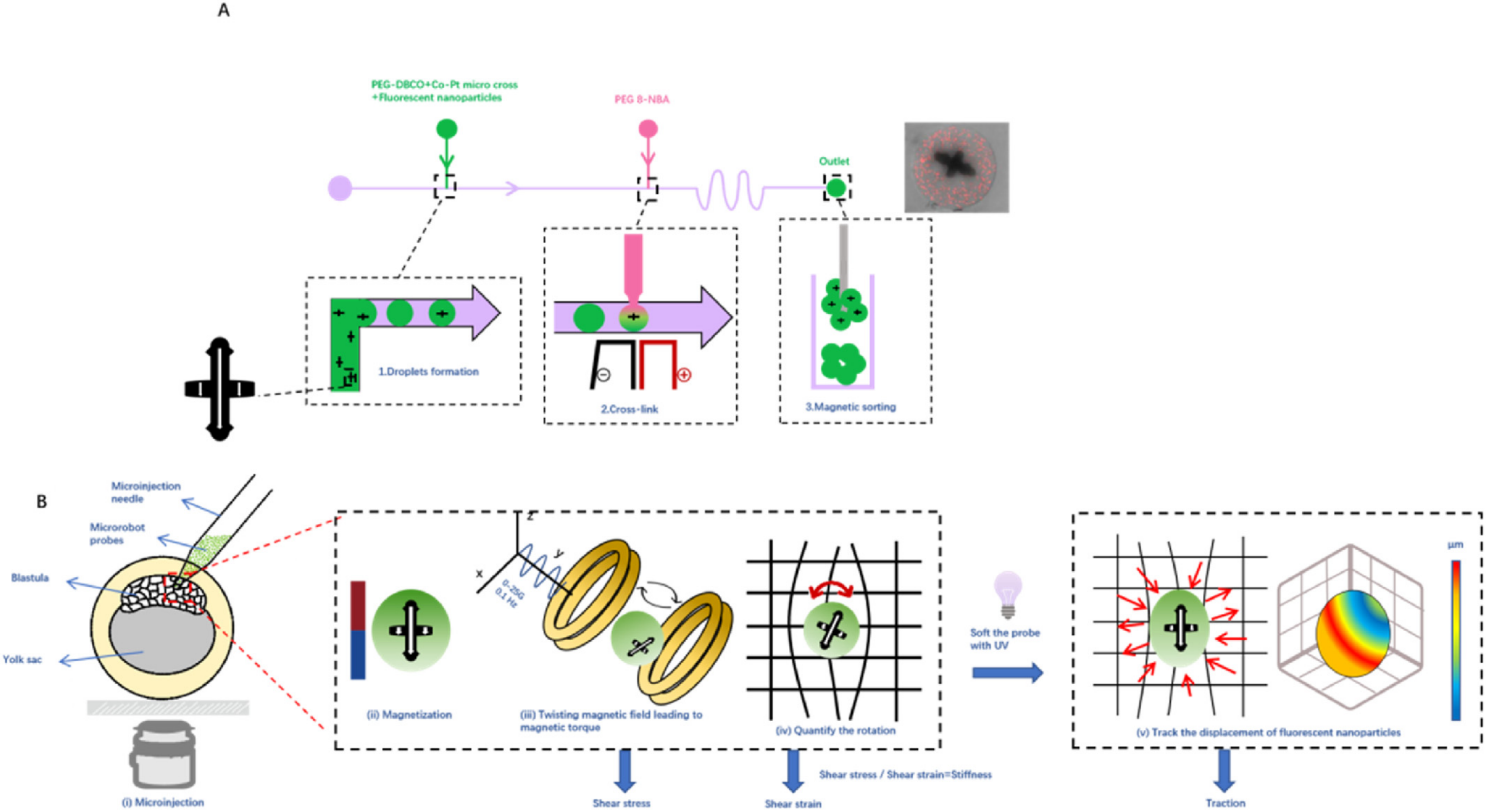
**A magnetic microrobot quantifies stiffness and traction forces in a zebrafish embryo**. A: Fabrication of microrobot probes by a microfluidic device. B: Microrobot probes are injected near the embryo's blastula tissues. The probes are permanently magnetized along the x-axis. A sinusoidal twisting field along the y-axis generates an oscillating magnetic torque in the z-direction. This torque causes the rigid probe to rotate (red arrow), deforming nearby cells/tissues for stiffness measurement. To measure 3D traction forces exerted onto the probe, the microrobot probe is softened using 405-nm UV light to measure the 3D traction forces. This figure is modified from Reference 73.

A miniaturized magnetic robot has been engineered to measure both mechanical parameters.^73^ Simultaneous measurements of both tractions and stiffness necessitate to develop tools that are rigid enough to measure stiffness and flexible enough to deform for traction determination (Figs. 1G and 3). In the current endeavor, a platinum-cobalt alloy cross that is ferromagnetic is embedded within a polyethylene glycol (PEG) gel, which photodegrades upon UV radiation exposure.^74^ The microprobe rotates in response to a magnetic field to measure embryo or 3D tissue stiffness. Upon a brief exposure to UV radiation, the PEG gel becomes soft and the same probe deforms under the cellular forces from the nearby cells. Using the strain field on the probe and knowing the probe's elastic properties, one can evaluate cellular tractions. This technique has been utilized to investigate the modulus and traction forces *in vitro* within colonies of tumor-repopulating cells and *in vivo* zebrafish embryos. Additionally, it reveals shear traction forces and substantial normal traction oscillations in zebrafish embryos *in vivo*. The method, however, faces limitations of not being able to make repeated measurements of stiffness and tractions. Future material advancements are required to enable repeated measurements of tractions and stiffness. Additionally, the magnetizable microprobe (50 ​μm) is too large for mouse embryos (100 ​μm), indicating the need for further miniaturization of the device. A recent method, which shines a laser to illuminate living tissues and measures thermal motion of native tissue structures over a wide range of frequencies, uses the fluctuation of speckles to extract moduli of tissues.^75^ However, the modulus of the bone obtained from this approach is only ∼10^6^ ​Pa, 3 orders of magnitude lower than published values using conventional approaches. The substantial underestimation of modulus by this approach is most likely due to the assumption that all processes in living tissues are thermal energy driven in calculating the tissue moduli. In addition, it is not known if this method could be useful in employing alterations in speckle fluctuations as an index for relative changes in tissue moduli.

#### 3.2.6 Significance of 3D force sensors

Three-dimensional force sensors provide substantial benefits for characterizing traction forces both *in vitro* and *in vivo*. Customizable with diverse materials, these sensors accommodate a spectrum of force measurements suitable for cells with varying stiffness. These tools enable researchers to study interactions between tissues, cells, and their environment. For example, they reveal uneven force patterns within tumor spheres.^76^ These findings also show that directional stress changes correlate with biological factors like cell division rates. Moreover, the oil droplet technique has permitted the initial quantitative assessment of embryonic tensile traction forces.^52^ Studies employing alginate elastomeric microspheres^59^ and magnetic microprobes^73^ have further shown that zebrafish embryos experience varying predominant normal forces at different developmental stages. These methodological advancements are essential for elucidating the spatiotemporal dynamics of forces during embryogenesis and cancer progression. We have summarized the key traction force measurement techniques from 2D to 3D in Table 1.

**Table1:** Comparisons among Traction Measurement Techniques in Mechanobiology.

Technique	Force Range	Applications	Advantages	Limitations	Ref
2D traction force microscopy (TFM)	nN to μN	Cell migration, adhesion	High spatiotemporal resolution; established protocols for substrate preparation and analysis.	Limited to 2D environment; cannot capture out-of-plane forces.	17, 18, 19, 20,23
3D traction force microscopy (3D TFM)	pN to μN	3D cell cultures, tumor spheroids, tissue engineering	Mimics physiological 3D microenvironments.	Computationally intensive; limited application *in vivo*.	41,45, 46, 47, 48
DNA force probe	pN to nN	Single-molecule forces (integrins, TCR, Notch, etc)	Molecular-level sensitivity; dynamic force tracking in real time; modular design for ligand-specific measurements.	Restricted to tensile forces; no force directions.	29, 30, 31, 32, 33, 34, 35, 36, 37, 38,91, 92, 93,103, 104, 105
Oil microdroplet force probes	Pa-kPa	Live embryonic tissues	Noninvasive for *in vivo* measurements; compatible with opaque tissues; quantifying only anisotropic stresses.	No volume changes of the probe under stress; not being able to detect hydrostatic stress and complete deviatoric stress.	52
3D elastic force sensors	10 ​Pa to kPa	Tumor generated forces; multicellular stress mapping	Quantifying both normal and shear stresses; biocompatible for long-term culture.	Requiring complex image analysis for 3D displacement fields.	54, 55, 56, 57, 58,76
Elastic microgel force probe	0.5–1.2 ​kPa	In vivo traction in embryos, tumors	Real-time monitoring of forces exerted by cell growth.	Requiring imaging equipment.	59
FRET-based force sensors	pN to nN	Focal adhesions, cell–cell junctions, measuring force transmission across the nuclear envelope	Non-invasive and genetically encodable. Real-time monitoring of molecular tension.	Limited to pre-defined molecular pairs; requiring genetic modification; sensitive to photobleaching and background noise.	63, 64, 65, 66, 67, 68, 69, 70
Magnetic microrobots	Pa-kPa	In vivo stiffness and traction quantification with the same probe in embryos and tumors	Measurement of stiffness and traction at the same location; applicable to complex 3D environments.	Large probe size (>50 ​μm); limited spatial resolution; requiring specialized equipment.	73
Infrared nanosensors	pN to μN	Immune cell interactions, single-cell mechanics	Penetrating scattering tissues (e.g., tumors). High-throughput potential.	Yet-to-be-validated in 3D *in vivo* settings; requiring advanced imaging systems.	111
Ferrofluid Microactuators	mPa to kPa	Tumor mechanical heterogeneity	Mapping spatially varying viscoelasticity; combining mechanical actuation and sensing.	Low spatial resolution; potential interference with tissue mechanics.	60,72

Collectively, the evolution of 3D force sensors has paved the way for exploring the mechanistic foundations of tissue morphogenesis and disease. Ranging from micro spheroids to compliant microparticles and bioprinted hydrogels, these technologies provide a comprehensive toolkit for investigating the complex mechanics of living tissues. The integration of these methodologies will be instrumental in deciphering the intricate interplay between force and function in biological systems. Using microsensors *in vivo* is a considerable gain, although their long-term viability and potential impacts on cellular stress distribution and interactions have not been thoroughly investigated.^77^ In conclusion, the goal is to enhance optical imaging capabilities further, to develop multifunctional sensors for mechanical property detection, and to develop sensors with good mechanical properties, high sensitivity, good biocompatibility *in vivo*, and adaptability to different force magnitudes, eventually allowing *in vivo* monitoring of forces during *in vivo* activities.

## 4 Applications of traction forces measurements in mechanomedicine

### 4.1 Diagnosis and treatment of diseases using traction force methodology

Traction forces are critical for diagnosing and treating diseases. Numerous methods now measure these forces at the cellular level, improving our understanding of their role in pathology. A multiple particle tracking method was employed to estimate the mechanical activity of epithelial cells,^78^ shedding light on cell migration, a process critical in disease progression. High-resolution traction force microscopy techniques,^79^ including substrate fabrication and imaging procedures, were detailed to delve into cell–matrix interactions. A traction force microscope method for three-dimensional measurement of weak traction forces of mitotic cells was advanced,^80^ providing insights into cell–matrix interactions during cell division. CONTRAX,^81^ a pipeline for tracking the contractile dynamics of single hiPSC-derived cardiomyocytes, was introduced, demonstrating the approach's utility in studying cardiomyopathies and cardiotoxicity. Collectively, these studies enhance our understanding of cellular mechanics, disease mechanisms, and potential treatment strategies. A nanotube-based method quantified mechanical behavior in single cancer cells,^82^ potentially reporting metastatic processes. Direct adhesion/traction force measurements between leukemia cells and their microenvironment^83^ showed how phorbol 12-myristate 13-acetate (PMA) treatment alters cell migration. Scanning ion conductance microscopy and traction force microscopy were combined to identify a correlation between local stiffness and traction force density in cancer cells,^84^ suggesting alterations in these interplays compared to healthy cells. These studies collectively contribute to the growing body of knowledge on traction forces in cancer research and their implications for diagnosis and treatment. Despite the acknowledged significance of stiffness and interstitial fluid pressure as diagnostic and prognostic indicators of cancer,^85^ there is little understanding regarding the therapeutic application of solid stress (the solid stress is defined as the cell growth induced stress onto the extracellular matrix). The investigators discovered that differences in stiffness and solid stress are distinct markers of mechanical abnormalities in malignancies, with primary and metastatic tumors exhibiting similar stiffness values but notably different solid stress levels.^12^ The potential therapeutic use of these findings is significant; for instance, recombinant hyaluronidase has been shown to enhance the effectiveness of chemotherapy by degrading hyaluronic acid and alleviating mechanical pressures on tumor vasculature.^86^ Quantitative stress measurement techniques could facilitate the development and testing of more tailored mechanical force medicines. The needle biopsy approach simplifies the acquisition of stress data on *in vivo* tumors, offering a glimpse into a future where mechanical data, such as stress and stiffness of different tumors, could inform the development of mechanical force drugs tailored to match the varying degrees of stress and stiffness identified in a patient's biopsy, thereby enabling more precise treatment selection.

### 4.2 Cellular tractions in immune cells

Immune cells can sense and respond to biophysical cues, including dynamic forces and spatial features, during their development, activation, differentiation, and expansion.^87^^,^^88^ The execution of their functions relies on the generation of mechanical forces by immune cells.^89^ For example, natural killer (NK) cells migrate through dense tissues at speeds of several micrometers per minute.^90^ These observations highlight the need for quantitative methods to measure the spatiotemporal distribution of mechanical forces produced by immune cells. Therefore, there is an urgent need for quantitative methods to detect the spatiotemporal distribution of mechanical forces produced by immune cells. The development of force measurement techniques has advanced our understanding of immune cells.

Immune cell recognition of antigens is the first step in their function, and with the development of technology, it has been discovered that the force generated by the T cell receptor (TCR) during antigen recognition is in the pN range.^91^^,^^92^ DNA tension probes have further revealed how LFA-1/ICAM-1 interactions regulate TCR-triggered T cell activation.^93^ Additionally, antigen stimulation intensity directly influences immune cell killing efficacy.^94^^,^^95^ Mechanical force stimulation can activate the BCR receptors of B cells, using a double-stranded DNA-based tension measurement system as a preset mechanical force measurement tool, with a range from 12 to 56 ​pN (pN).^96^^,^^97^ DNA-based molecular sensors are also used to measure the mechanical forces generated by B cells during antigen extraction at immune synapses.^98^, ^99^, ^100^ The traditional Traction Force Microscopy (TFM) technique is combined with a confocal live-cell imaging system; it is shown that B cells generate centripetal traction forces of 10–20 ​nN during the spreading-contraction activation process on an elastic substrate that mimics the stiffness of antigen-presenting cells (APCs) *in vivo*.^101^^,^^102^ Quantifying the mechanics of T cell receptor (TCR) at immune cell–cell junctions using DNA origami tension sensors (DOTS) has been a significant advancement in the field of immunology.^103^^,^^104^ From the atomic level to the cellular level, the scale revelation of how biological forces dynamically regulate the conformational changes of antigen-presenting molecules (pMHC-I) to determine T cell receptor (TCR) antigen recognition can be detected by Biomembrane Force Probe, providing key theoretical foundations and technical support for the prediction of tumor neoantigens, the development of immune disease drugs, and the optimization of clinical immune treatment plans.^105^

Mechanotransduction critically influences immune cell activation and differentiation. During immune synapse formation, cytoskeletal remodeling occurs, with mechanical signals playing key roles in immune responses.^106^ The formation of immune synapses is crucial for the function of T cells. Researchers have developed a Microparticle Traction Force Microscopy (TFM) technique, which involves the synthesis of uniform, deformable, and tunable hydrogel microparticles to reveal the dynamic changes in forces within cytotoxic T cell immune synapses.^71^ In the process of immune killing, cytotoxic T cells (CTLs) enhance the destruction of target cells by exerting mechanical forces within the immune synapse.^107^ Super-resolution traction force microscopy has been used to compare the immune synapses formed by CTLs with other T cell subsets and macrophages. Immune cells exhibit specific biomechanical characteristics when performing cytotoxic functions, which modulate immune responses through the application of force.^108^ Traction force microscopy has revealed that blocking the mechanical sensor PIEZO1 in T cells can enhance their traction force, thereby enhancing their cytotoxicity against tumor cells.^109^ Inhibition of non-muscle myosin II can increase the traction force exerted by T cells and enhance their cytotoxicity against tumors.^110^ Recently infrared nanosensors have been developed that can measure piconewton to micronewton forces.^111^ However, it is not clear if these sensors can quantify cellular tractions in 3D.

## 5 Conclusions

Cells respond to various external environmental stimuli and generate tractions that regulate various cellular processes. Precise quantification of these forces with high spatiotemporal resolution is indispensable for deciphering mechanobiological mechanisms and for advancing clinical diagnostics and therapeutics. Existing force sensors can measure 3D tractions *in vivo*. However, no single technique provides a universal solution. Each approach has its advantages and limitations. The rapid evolution of artificial intelligence (AI) has catalyzed a paradigm shift in biological research, spurring subcellular mechanics interrogation and dynamic behavior analysis. Platforms utilizing Generative Adversarial Networks (GANs) for molecular dynamics simulations have made it possible to precisely design fluorescent probes less than 10 ​μm. An example is the nitrogen-vacancy diamond probe. These AI-optimized probes offer subcellular positioning accuracy below 5 ​nm. This is an order of magnitude improvement over traditional methods. The enhanced accuracy improves the specificity of probe-target binding.^112^ Researchers have also developed an automated system for multidimensional single-particle tracking in living cells. This system uses deep learning to track individual particles within cells automatically. It collects and analyzes the dynamic processes inside cells with greater efficiency.^113^ These innovations highlight AI's potential in biological research. Future developments in measurement technology should focus on improving spatiotemporal resolution, achieving high-throughput measurements, and enhancing their applicability in medical and clinical settings.

## CRediT authorship contribution statement

**Xinman Chen:** Writing – original draft. **Chenyang Ji:** Writing – original draft. **Xi Liu:** Writing – original draft. **Ning Wang:** Writing – review & editing, Writing – original draft, Funding acquisition, Conceptualization. **Fuxiang Wei:** Writing – review & editing, Writing – original draft, Funding acquisition. **Junwei Chen:** Writing – review & editing, Writing – original draft, Funding acquisition.

## Ethical approval

This study does not contain any studies with human or animal subjects performed by any of the authors.

## Declaration of competing interest

The authors declare no financial interests/personal relationships which may be considered as potential competing interests.
